# Fluid Biomarkers in Individuals at Risk for Genetic Prion Disease up to Disease Conversion

**DOI:** 10.1212/WNL.0000000000209506

**Published:** 2024-06-19

**Authors:** Sonia M. Vallabh, Meredith A. Mortberg, Shona W. Allen, Ashley C. Kupferschmid, Pia Kivisakk, Bruno L. Hammerschlag, Anna Bolling, Bianca A. Trombetta, Kelli Devitte-McKee, Abaigeal M. Ford, Lauren E. Sather, Griffin Duffy, Ashley Rivera, Jessica Gerber, Alison J. McManus, Eric V. Minikel, Steven E. Arnold

**Affiliations:** From the McCance Center for Brain Health (S.M.V., M.A.M., E.V.M.) and Department of Neurology (S.M.V., M.A.M., S.W.A., A.C.K., P.K., B.L.H., A.B., B.A.T., K.D.-M., A.M.F., L.E.S., G.D., A.R., J.G., A.J.M., E.V.M., S.E.A.), Massachusetts General Hospital, Boston; Stanley Center for Psychiatric Research (S.M.V., M.A.M., E.V.M.), Broad Institute of MIT and Harvard, Cambridge; and Department of Neurology (S.M.V., P.K., E.V.M., S.E.A.), Harvard Medical School, Boston, MA.

## Abstract

**Objectives:**

To longitudinally characterize disease-relevant CSF and plasma biomarkers in individuals at risk for genetic prion disease up to disease conversion.

**Methods:**

This single-center longitudinal cohort study has followed known carriers of *PRNP* pathogenic variants at risk for prion disease, individuals with a close relative who died of genetic prion disease but who have not undergone predictive genetic testing, and controls. All participants were asymptomatic at first visit and returned roughly annually. We determined *PRNP* genotypes, measured NfL and GFAP in plasma, and RT-QuIC, total PrP, NfL, T-tau, and beta-synuclein in CSF.

**Results:**

Among 41 carriers and 21 controls enrolled, 28 (68%) and 15 (71%) were female, and mean ages were 47.5 and 46.1. At baseline, all individuals were asymptomatic. We observed RT-QuIC seeding activity in the CSF of 3 asymptomatic E200K carriers who subsequently converted to symptomatic and died of prion disease. 1 P102L carrier remained RT-QuIC negative through symptom conversion. No other individuals developed symptoms. The prodromal window from detection of RT-QuIC positivity to disease onset was 1 year long in an E200K individual homozygous (V/V) at PRNP codon 129 and 2.5 and 3.1 years in 2 codon 129 heterozygotes (M/V). Changes in neurodegenerative and neuroinflammatory markers were variably observed prior to onset, with increases observed for plasma NfL in 4/4 converters, and plasma GFAP, CSF NfL, CSF T-tau, and CSF beta-synuclein each in 2/4 converters, although values relative to age and fold changes relative to individual baseline were not remarkable for any of these markers. CSF PrP was longitudinally stable with mean coefficient of variation 9.0% across all individuals over up to 6 years, including data from converting individuals at RT-QuIC-positive timepoints.

**Discussion:**

CSF prion seeding activity may represent the earliest detectable prodromal sign in E200K carriers. Neuronal damage and neuroinflammation markers show limited sensitivity in the prodromal phase. CSF PrP levels remain stable even in the presence of RT-QuIC seeding activity.

**Clinical Trials Registration:**

ClinicalTrials.gov NCT05124392 posted 2017-12-01, updated 2023-01-27.

## Introduction

Prion disease exhibits striking biomarker signatures at the symptomatic stage,^1-4^ but data about presymptomatic changes are limited (Supplementary Background, eAppendix 1). Neurodegeneration and neuroinflammation markers may rise 2 years before onset in slowly progressive subtypes such as P102L, but only months before onset in rapidly progressive subtypes D178N and E200K,^3,5^ mirroring disease duration.^6^ Prion seeding activity has been detected by RT-QuIC in CSF before onset in E200K individuals, but the prognostic value is unknown. Here, we report fluid biomarker trajectories associated with 4 disease onsets over 6 years in a longitudinal natural history of individuals at risk for genetic prion disease.

## Methods

### Standard Protocol Approvals, Registrations, and Patient Consents

Participants provided written consent. The study was approved by the MGB Institutional Review Board (2017P000214). Assay validation used MIND Tissue Bank (2015P000221) samples. This study is registered with ClinicalTrials.gov (NCT05124392).

### Study Participants

This previously described^7^ cohort study at Massachusetts General Hospital includes asymptomatic individuals with or without pathogenic *PRNP* variants (Table 1; eFigure 1; eMethods), invited to contribute blood and CSF approximately annually. Data presented here include data previously reported.^7,8^ We performed *PRNP* genotyping on all individuals including those who did not know their own genetic status; steps taken to prevent self-identification are described in eMethods.

**Table 1:** Demographic Characteristics of the Cohort

Group	N	Sex	Age (y)	Follow-up (y)	Total visits	CSF samples	Plasma samples	Pathogenic variants
Pathogenic variant carrier	41	13M/28F	47.5 ± 14.0	2.0 ± 1.9	126	104	109	6 P102L 7 D178N 22 E200K 6 other
Control	21	6M/15F	46.1 ± 13.3	1.4 ± 1.5	57	51	51	21 none

“Age” represents age last seen, follow-up is years from first visit to last visit, and both are represented by mean ± SD.

### Biomarker Assays

Biomarker assays used were RT-QuIC (IQ-CSF protocol),^9^ PrP ELISA^8^ (eFigure 2), Simoa (Quanterix) GFAP, and Ella (Bio-Techne) NfL, T-tau (eFigure 3), and β-syn (eFigure 4), see eMethods.

### Statistical Analysis

Biomarker relationships with age and genotype were assessed by log-linear regression; curve fits shown in figures are the separate best fits for pathogenic variant carriers and for controls, while *p* values are for the effect of carrier status in a combined model: lm(log(value) ∼ age + carrier). For details of RT-QuIC data analysis, see eMethods. *p* values < 0.05 were considered nominally significant. Analyses were conducted in R 4.2.0.

### Data Availability

Full biomarker values for all participants will be made available to qualified investigators with ethical approval and a data use agreement upon request. Source code, summary statistics for all participants, and individual biomarker values for converting participants are freely available at github.com/ericminikel/mgh_prnp_freeze2.

## Results

Sixty-two participants completed at least 1 study visit. 41 harbored pathogenic *PRNP* variants (“carriers”), and 21 were negative (“controls”"). Groups were well-matched, and distribution of *PRNP* genotypes was consistent with pathogenic variant prevalence^10^ (Table 1). We collected 155 CSF samples and 160 plasma samples. From July 2017 to February 2023, 4 carriers converted to active disease (N = 3 E200K, N = 1 P102L). We performed fluid biomarker analyses on samples from both converters (eTable 1) and nonconverting carriers and controls (eTable 2).

**Figure 1:**
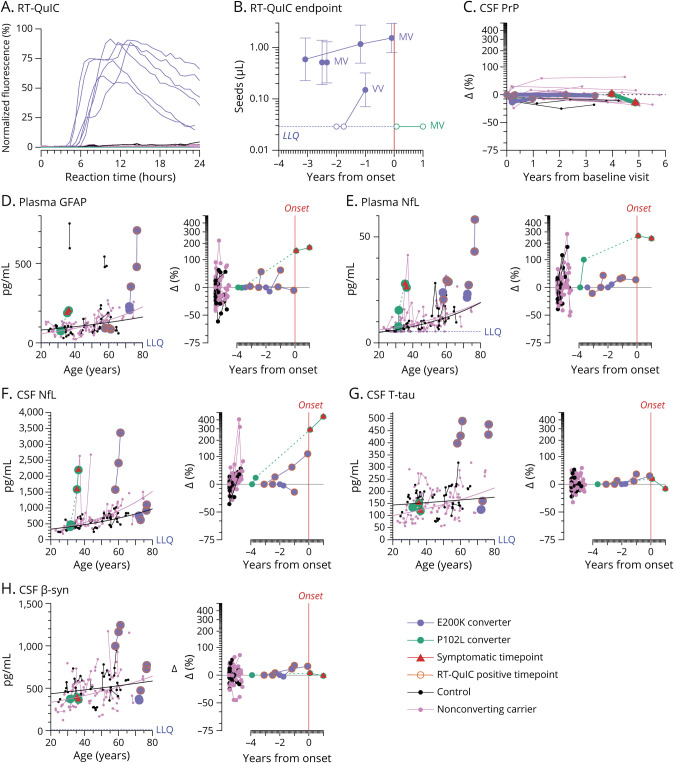
Fluid Biomarker Changes in the Cohort (A) RT-QuIC kinetic curves for N = 149 CSF samples tested (98 from carriers and 51 from controls), showing 6 positive CSF samples (each with 4/4 replicates positive). (B) RT-QuIC endpoint titration of N = 10 CSF samples from 4 unique individuals who developed disease, including the 6 positive CSF samples from 3 E200K converters, with codon 129 genotypes of converters indicated. (C) CSF PrP concentrations represented as changes (∆) relative to individual baseline, shown for the 4 converters plus all individuals with at least 3 years between first and last available CSF sample. N = 76 CSF samples from 19 unique individuals, see eTable 4. (D–H) Biomarkers plasma GFAP (N = 158 samples from 61 unique individuals) (D), plasma NfL (N = 160 samples from 62 unique individuals) (E), CSF NfL (N = 155 samples from 60 unique individuals) (F), CSF T-tau (N = 151 samples from 60 unique individuals) (G), and CSF β-syn (N = 150 samples from 60 unique individuals) (H) are represented by 2 views each. Left: individual age vs absolute concentration in pg/mL, with sequential samples from the same individual connected by thin lines, while thicker lines represent the separate log-linear best fit curves for controls and for nonconverting carriers. Right: years from disease onset vs change (∆) relative to individual baseline in converters, with the same for controls and for nonconverting carriers shown on a separate x-axis. Dashed lines connect timepoints before and after symptom onset. For further breakdown and statistics, see eTable 5.

Testing of longitudinal CSF samples by RT-QuIC identified 6 positive samples (Figure 1A), all of which belonged to the 3 E200K individuals who had developed disease and died. Each CSF sample from these individuals was re-tested by endpoint dilution^5,9^ to determine the prion titer (prion seeds per µL). Of these 3 E200K individuals, 2 *PRNP* codon 129 heterozygotes (each cis-129M, trans-129V) were already RT-QuIC positive at first lumbar puncture (2.5 and 3.1 years before onset) and prion titer in CSF did not appreciably rise thereafter (Figure 1B). One homozygote (V/V) was negative at the first 2 visits, became RT-QuIC positive on study and subsequently became symptomatic 1 year later.

CSF total PrP levels varied between individuals and were lower in carriers (eFigure 2, eTable 3) but were longitudinally stable in each individual out to 6 years (Figure 1C, eTable 4), including samples taken after RT-QuIC positivity.

Plasma GFAP, a marker of reactive astrogliosis, was high relative to age in 2/4 converters, but change from individual baseline was unremarkable compared to controls and nonconverters (Figure 1D). Plasma NfL appeared high and increased in all 4 converters, but not outside the range of nonconverters and controls (Figure 1E, eTable 5). CSF NfL, CSF T-tau, and CSF β-syn were each elevated in 2/4 converters and normal in 2/4 (Figure 1, F–H, eTable 5); different converting individuals were high for different markers.

## Discussion

Here we describe fluid biomarker profiles in a longitudinal cohort of carriers of pathogenic *PRNP* variants, including 4 individuals who converted to active disease. As before,^3,5,7^ at any given time, cross-sectionally, most carriers of prion disease-causing variants lacked any detectable molecular sign of the disease. Our data support the hypothesis that CSF prion seeding activity assayed by RT-QuIC may represent the first detectable change in E200K carriers. We did not detect seeding activity in the CSF of a P102L converter, consistent with RT-QuIC's lower sensitivity in the context of disease subtypes hypothesized to exhibit lower intrinsic seeding capacity.^1^ We observed longer prodromal positivity in 2 codon 129 M/V heterozygotes than in 1 V/V homozygote; if replicated in larger cohorts, this difference would mirror the longer disease duration after onset in heterozygotes.^11^

Soluble PrP in CSF is reduced in symptomatic prion disease patients, presumably as a result of a disease sink process,^12^ and yet pharmacologic lowering of CSF PrP may be important as a drug activity biomarker for trials of PrP-lowering drugs, and has been proposed as a surrogate endpoint in prevention trials.^12^ Our data suggest CSF PrP does not decline prior to symptom onset, even in the presence of RT-QuIC positivity, suggesting its use in asymptomatic individuals will not be confounded.

Neuronal damage and neuroinflammation markers rise with age and may vary between individuals. Neither when normalized to age nor to individual baseline did any of these markers consistently provide distinctive signal in all 4 of our converting individuals relative to nonconverters and controls. Despite the excellent diagnostic utility of β-syn in discriminating prion disease from other rapidly progressive dementias,^2^ it was not more consistently elevated than CSF T-tau or CSF NfL in individuals proximate to conversion. While these markers may be useful as an adjunct, none is likely to provide the prognostic specificity of RT-QuIC. RT-QuIC, meanwhile, may offer just 1 year of advance signal in some E200K cases, and currently faces limited sensitivity to other subtypes. Assay improvement, biomarker discovery, and continued sample accrual will be vital to identifying additional prognostic markers, particularly for non-E200K subtypes. At any given time, most carriers appear nonprodromal; thus, in this rare disease, prodromal individuals are unlikely to be identified in sufficient numbers to power clinical trials. Primary prevention trials with inclusion based on genotype and CSF PrP as primary endpoint are one possibility,^12^ which would honor the outsize benefit of early treatment observed in animal models.^13^ Nonetheless, treatment of prodromal individuals could feature as a supportive arm and/or randomization off-ramp for carriers who develop a prodromal signature during a trial and enhancing our ability to identify prodromal states should be a research priority.

Our study has limitations. Four symptom onsets is a small absolute number from which to draw conclusions. Reflecting study enrollment and overall genotypic prevalence, our observed onsets are skewed toward E200K. Some annual visits were missed due to COVID-19. We did not collect emerging sample types such as nasal brushings^14^ or tears,^15^ and we did not perform MRI or ^18^FDG-PET. We used only standard RT-QuIC conditions^9^ and did not explore alternative recombinant PrP substrates such as bank vole PrP^5^ or E200K PrP,^15^ which might enhance sensitivity in certain genetic subtypes. Additional presymptomatic natural history work across multiple sites will be required to build confidence in our observations.

## Appendix Authors

**Table:**

Name	Location	Contribution
Sonia M. Vallabh, JD, PhD	McCance Center for Brain Health and Department of Neurology, Massachusetts General Hospital, Boston; Stanley Center for Psychiatric Research, Broad Institute of MIT and Harvard, Cambridge; Department of Neurology, Harvard Medical School, Boston, MA	Drafting/revision of the manuscript for content, including medical writing for content; major role in the acquisition of data; study concept or design; analysis or interpretation of data
Meredith A. Mortberg, BS	McCance Center for Brain Health and Department of Neurology, Massachusetts General Hospital, Boston; Stanley Center for Psychiatric Research, Broad Institute of MIT and Harvard, Cambridge, MA	Drafting/revision of the manuscript for content, including medical writing for content; major role in the acquisition of data
Shona W. Allen, BS	Department of Neurology, Massachusetts General Hospital, Boston	Major role in the acquisition of data
Ashley C. Kupferschmid, BS	Department of Neurology, Massachusetts General Hospital, Boston	Major role in the acquisition of data
Pia Kivisakk, MD, PhD	Department of Neurology, Massachusetts General Hospital; Department of Neurology, Harvard Medical School, Boston, MA	Drafting/revision of the manuscript for content, including medical writing for content; major role in the acquisition of data
Bruno L. Hammerschlag, BS	Department of Neurology, Massachusetts General Hospital, Boston	Major role in the acquisition of data
Anna Bolling, BS	Department of Neurology, Massachusetts General Hospital, Boston	Major role in the acquisition of data
Bianca A. Trombetta, BS	Department of Neurology, Massachusetts General Hospital, Boston	Major role in the acquisition of data
Kelli Devitte-McKee, MSN	Department of Neurology, Massachusetts General Hospital, Boston	Major role in the acquisition of data
Abaigeal M. Ford, BS	Department of Neurology, Massachusetts General Hospital, Boston	Major role in the acquisition of data
Lauren E. Sather, MSc	Department of Neurology, Massachusetts General Hospital, Boston	Major role in the acquisition of data
Griffin Duffy, BS	Department of Neurology, Massachusetts General Hospital, Boston	Major role in the acquisition of data
Ashley Rivera, BS	Department of Neurology, Massachusetts General Hospital, Boston	Major role in the acquisition of data
Jessica Gerber, MS	Department of Neurology, Massachusetts General Hospital, Boston	Major role in the acquisition of data
Alison J. McManus, DNP	Department of Neurology, Massachusetts General Hospital, Boston	Major role in the acquisition of data
Eric V. Minikel, PhD	McCance Center for Brain Health and Department of Neurology, Massachusetts General Hospital, Boston; Stanley Center for Psychiatric Research, Broad Institute of MIT and Harvard, Cambridge; Department of Neurology, Harvard Medical School, Boston, MA	Drafting/revision of the manuscript for content, including medical writing for content; study concept or design; analysis or interpretation of data
Steven E. Arnold, MD	Department of Neurology, Massachusetts General Hospital; Department of Neurology, Harvard Medical School, Boston, MA	Drafting/revision of the manuscript for content, including medical writing for content; major role in the acquisition of data; study concept or design
